# Paracrine brassinosteroid signaling at the stem cell niche controls cellular regeneration

**DOI:** 10.1242/jcs.204065

**Published:** 2018-01-15

**Authors:** Fidel Lozano-Elena, Ainoa Planas-Riverola, Josep Vilarrasa-Blasi, Rebecca Schwab, Ana I. Caño-Delgado

**Affiliations:** 1 Department of Molecular Genetics, Centre for Research in Agricultural Genomics (CRAG) CSIC-IRTA-UAB-UB, Barcelona E-08193, Spain; 2 Cold Spring Harbor Laboratory, 1 Bungtown Road, Cold Spring Harbor, NY 11724, USA

**Keywords:** Brassinosteroid, Quiescent center, Cell division, Stem cell, DNA damage, Paracrine

## Abstract

Stem cell regeneration is crucial for both cell turnover and tissue healing in multicellular organisms. In Arabidopsis roots, a reduced group of cells known as the quiescent center (QC) act as a cell reservoir for surrounding stem cells during both normal growth and in response to external damage. Although cells of the QC have a very low mitotic activity, plant hormones such as brassinosteroids (BRs) can promote QC divisions. Here, we used a tissue-specific strategy to investigate the spatial signaling requirements of BR-mediated QC divisions. We generated stem cell niche-specific receptor knockout lines by placing an artificial microRNA against BRI1 (BRASSINOSTEROID INSENSITIVE 1) under the control of the QC-specific promoter WOX5. Additionally, QC-specific knock-in lines for BRI1 and its downstream transcription factor BES1 (BRI1-EMS-SUPPRESOR1) were also created using the WOX5 promoter. By analyzing the roots of these lines, we show that BES1-mediated signaling cell-autonomously promotes QC divisions, that BRI1 is essential for sensing nearby inputs and triggering QC divisions and that DNA damage promotes BR-dependent paracrine signaling in the stem cell niche as a prerequisite to stem cell replenishment.

## Abstract (precis)

**Summary:** Quiescent cell division at the stem cell niche is activated by exogenous brassinosteroid signals and points to the brassinosteroid hormone as a limiting factor for stem cell division.

## INTRODUCTION

Brassinosteroids (BRs) are plant steroid hormones that were originally discovered in *Brassica napus* pollen for their ability to promote growth when exogenously applied to other vascular plants (Mitchell et al., 1970). Impaired BR biosynthesis or signaling causes reduced organ growth and abnormal development, and thereby limits plant fertility and yield (Li and Chory, 1997; Wei and Li, 2016). Despite parallels between the functions of plant and animal steroid hormones (Li and Chory, 1997; Thummel and Chory, 2002), substantial differences exist with respect to their perception and signal transduction mechanisms. Whereas animal steroid perception is mainly mediated by transcription factors inside the cell (Aranda and Pascual, 2001), plant steroids are perceived by leucine-rich repeat (LRR) receptor kinases located at the plasma membrane (Kim and Wang, 2010).

BR signaling is initiated by the direct binding of the steroid molecule to a 93 amino acid region located within the extracellular domain of the LRR receptor kinase BRI1 (BRASSINOSTEROID INSENSITIVE 1) (Hothorn et al., 2011; Kinoshita et al., 2005; Wang et al., 2001). Upon BR binding, the heterodimerization of BRI1 with BAK1 (BRI1-ASSOCIATED RECEPTOR KINASE 1) is enhanced, and a cytoplasmic cascade of phosphorylation and dephosphorylation events is initiated (Li and Nam, 2002; Russinova et al., 2004). These events lead to the degradation of BIN2 (BRASSINOSTEROID INSENSITIVE 2) kinase (Li and Nam, 2002; Peng et al., 2008), and a consequential increase in the dephosphorylated forms of the BZR1 (BRASSINAZOLE RESISTANCE 1) (Wang et al., 2002) and BES1 (BRI1-EMS-SUPRESSOR 1) (Yin et al., 2002) transcription factors. Dephosphorylated BZR1 and BES1 are translocated into the nucleus where they modulate the transcription of thousands of genes by directly interacting with DNA and other transcription factors (He et al., 2002). In fact, BZR1 and BES1 are known to bind specific DNA sequences: the BR-response element (BRRE, CGTGC/TG) and E-boxes (CANNTG) (He et al., 2005; Sun et al., 2010; Yu et al., 2011). Furthermore, recent work has revealed that these transcription factors are subjected to post-transcriptional regulation in response to external stimuli such as light (Kim et al., 2014) and environmental stress (Nolan et al., 2017). In this way, BR-mediated transcriptional responses are also controlled by an additional regulatory layer.

In addition to BRI1, Arabidopsis contains three BRI1-like (BRL) receptor kinase homologues. Interestingly, however, only BRL1 and BRL3 (BRI1-LIKE 1 and 3) are functional BR receptors capable of binding the hormone (Cano-Delgado et al., 2004). Although BRI1 is present in the majority of plant cells (Friedrichsen and Chory, 2001), the BRL1 and BRL3 receptors are enriched in vascular tissues and the stem cell niche (Cano-Delgado et al., 2004; Fàbregas et al., 2013; Salazar-Henao et al., 2016).

**Fig. 1.**
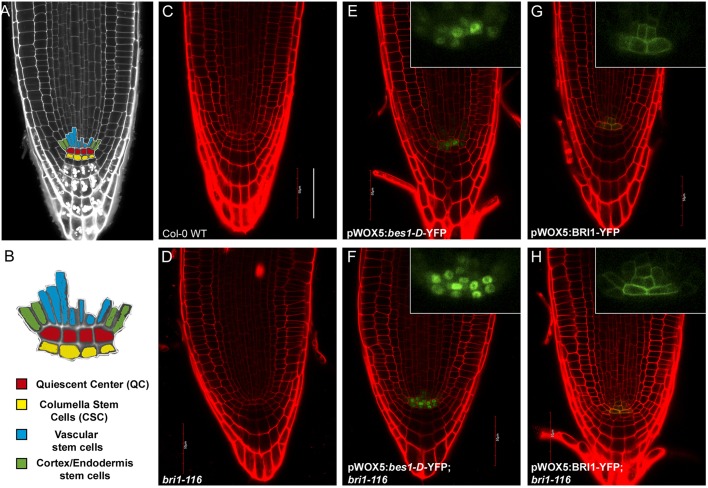
**The stem cell niche of Arabidopsis roots and QC-specific expression of BR pathway components.** (A) A stereotypical Arabidopsis WT primary root under confocal microscopy. The root stem cell niche is highlighted in color. (B) Detailed representation of the root stem cell niche. (C–H) Confocal images of 6-day-old WT and mutant Arabidopsis roots in control conditions. Green represents YFP-tagged pathway components. Red is PI counterstaining. Insets show the YFP channels at higher magnification. Scale bar: 50 µm.

By providing a continuous supply of precursor cells, stem cells are primarily involved in sustaining growth and replacing damaged tissues (Sablowski, 2004). Root stem cells, also known as initials, are located at the root apex and surround the quiescent center (QC) (Dolan et al., 1993) (Fig. 1A,B). The QC, which comprises a small group of cells with very low mitotic activity, not only acts as a cell reservoir for the surrounding actively dividing stem cells (Scheres, 2007; Dolan et al., 1993), but is also responsible for maintaining the stem cells in their undifferentiated state (Sabatini et al., 2003; van den Berg et al., 1997). However, upon cellular damage, the QC loses its quiescence and enters into a state of cell division to enable stem cell replenishment (Cruz-Ramírez et al., 2013; Heyman et al., 2013; Vilarrasa-Blasi et al., 2014).

Hormonal stimulation also plays an important role in governing cell division in the QC (Gonzalez-Garcia et al., 2011; Heyman et al., 2013; Zhang et al., 2010). For instance, BRs are known to promote both cell division in the QC and differentiation of the surrounding columella stem cells (Fàbregas et al., 2013; Gonzalez-Garcia et al., 2011; Vilarrasa-Blasi et al., 2014). More specifically, the ERF115 transcription factor, which is activated by BRs, promotes QC divisions and stem cell regeneration after DNA damage (Heyman et al., 2016, 2013). In contrast, BRAVO (BRASSINOSTEROIDS AT VASCULAR AND ORGANIZING CENTER), an R2R3-MYB transcription factor identified using cell-specific transcriptomics, acts as a repressor of QC divisions (Vilarrasa-Blasi et al., 2014). Interestingly, BRAVO is a direct transcriptional target of and interacts with the BR-regulated transcription factor BES1 at the protein level, forming a feedback loop that antagonistically regulates QC divisions (Vilarrasa-Blasi et al., 2014). Despite the importance of these transcription factors for locally safeguarding QC divisions, it is still unknown whether BR-regulated QC function is maintained in a cell-autonomous fashion or requires external signaling. Moreover, although BR receptors collectively modulate QC cell division and differentiation of surrounding stem cells under normal conditions (Fàbregas et al., 2013), the specific contribution of each receptor within the stem cell niche is not known.

These questions prompted us to investigate BR-mediated regulation of quiescence and its impact on stem cell regeneration after DNA damage at the local level. Accordingly, we used a tissue-specific approach in order to determine the ability of QC cells to integrate exogenous steroid signals. For this purpose, we specifically overexpressed two BR signaling components – the BRI1 membrane receptor and the BES1 transcription factor – in QC cells, and specifically knocked out BRI1 in the stem cell niche using an artificial microRNA (amiRNA) (Dolan et al., 1993; Schwab et al., 2006). Altogether, we demonstrate that: (1) active BES1 is necessary for cell-autonomous QC divisions; (2) the BR hormone itself (i.e. not the receptors) is the limiting factor for BR-induced QC divisions in the root apex; (3) BRI1 is required at the stem cell niche for mediating BR-dependent QC divisions; and (4) upon stem cell death, paracrine BR signaling is required for QC divisions. Overall, our results establish a hierarchy for the different BR receptors within the stem cell niche, indicating that under normal conditions the BRI1 receptor acts as the principal player controlling QC divisions, rather than its homologous.

## RESULTS

### Active BES1 promotes cell-autonomous QC division

We first wanted to elucidate whether the BR-induced division signals of the QC were transduced in a cell-autonomous manner through the canonical BR signaling cascade. To this end, we used the gain-of-function BES1 mutant, *bes1-D*, which is known to be constitutively active (Yin et al., 2002). Previously, we cloned *bes1-D* under the control of the promoter of the QC-specific gene *WOX5* (Sarkar et al., 2007), and fused YFP to its C-terminus (Vilarrasa-Blasi et al., 2014). This construct, pWOX5:*bes1-D*-YFP, was transformed into both Col-0 wild-type (WT) and the null BRI1 mutant *bri1-116* (Li and Chory, 1997) (Fig. 1C–F).

**Fig. 2.**
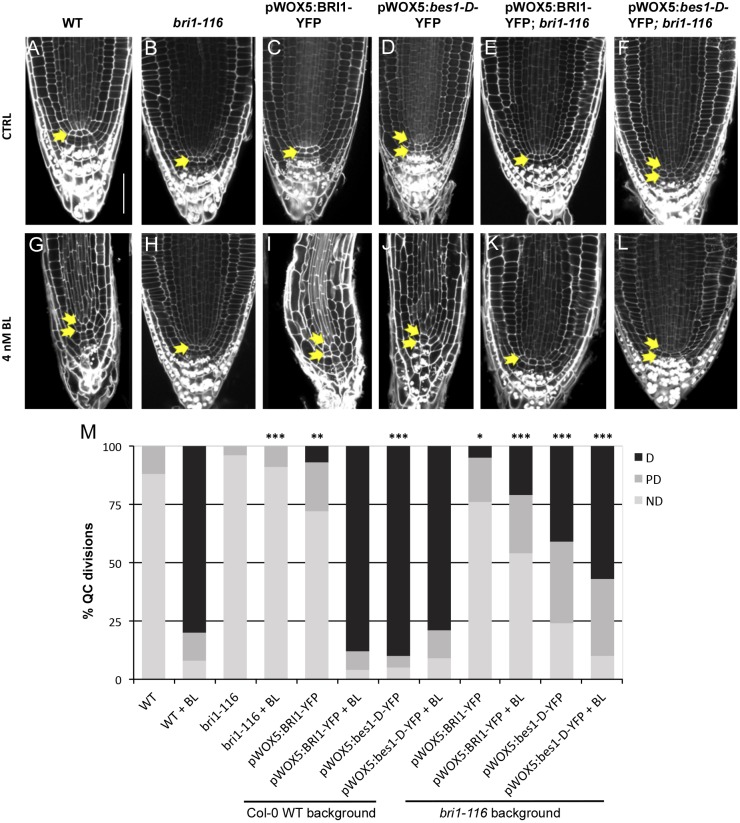
**The BR-regulated transcription factor BES1 promotes QC division in a cell-autonomous manner.** (A–F) Confocal images of fixed 6-day-old WT and mutant Arabidopsis roots in control conditions. (G–L) Root anatomy of 6-day-old seedlings grown in medium supplemented with 4 nM BL. Arrows indicate the number of QC cell layers identified. (M) Quantification of QC division rate. ND, QC non-divided; PD, QC partially divided; D, QC totally divided. Asterisks indicate statistically significant differences due to genotype, comparing against WT either in control or 4 nM BL conditions. Frequencies in QC divisions were assessed with a two-sided Fisher's test. Values for all pairwise comparisons are provided in Table S1. Data are generated from three independent replicates (*n*>21). *P<0.05, **P<0.01, ***P<0.005. Scale bar: 50 µm.

Confocal microscopy of 6-day-old roots revealed an increase in the number of QC divisions in both the WT and the *bri1-116* mutant upon expressing *bes1-D* under the *WOX5* promotor (Fig. 2A,D,F,M; Table S1). This indicates that active BES1 locally promotes division at the QC in a cell-autonomous manner. Interestingly, however, the QC division rates in the *bri1-116* background were lower than those in the WT background (Fig. 2M; Table S1), suggesting that BR signaling from surrounding tissues also participates in activation of QC divisions.

In addition, treatment of WT plants harboring the pWOX5:*bes1-D*-YFP construct with brassinolide (BL) did not result in a significant increase in cell division rates (Fig. 2D,J,M; Table S1). This is probably due to a saturated BRs signal contributed also by basal receptor-transduced signaling. Conversely, upon BL treatment, a significant increase in cell division rate was observed for the *bri1-116* plants that contained pWOX5:*bes1-D*-YFP (Fig. 2F,L,M; Table S1). This suggests that the signal is not saturated in these plants, and that the BRL receptors are also contributing factors.

### The local BR hormone level is the main limiting factor for QC division

Next, by introducing the pWOX5:BRI1-YFP transgene into both WT and *bri1-116* backgrounds, we evaluated the local contribution of the BRI1 receptor to QC division (Fig. 1C,E). As the *WOX5* promoter drives relatively high expression compared with the endogenous *BRI1* promoter, *WOX5*-controlled expression of the BRI1 receptor resulted in its local overexpression in the QC. Confocal images comparing BRI1 expression under its endogenous promoter (Geldner et al., 2007) with BRI1 expression in the pWOX5:BRI1-YFP lines are shown in Fig. S1.

When BRI1 is locally overexpressed using the *WOX5* promoter, a small increase in QC division rate was observed in both the WT and the *bri1-116* backgrounds (Fig. 2C,E,M; Table S1). This increase, however, was substantially smaller than that observed upon expression of *bes1*-D using the same promoter (Fig. 2D,F,M; Table S1). Upon application of exogenous BL, we observed a dramatic increase in the QC division rate for those plants expressing pWOX5:BRI1-YFP in the WT background but not in the *bri1-116* background (Fig. 2C,E,I,K,M; Table S1). This implies that BRI1 signaling in the QC alone is not sufficient to promote QC divisions, but rather additional external signaling is required. The fact that overexpression of BRI1 in the QC did not result in a large increase in QC division until exogenous BL was applied, indicates that the BR hormone itself is the limiting factor of QC division. Furthermore, only after applying BL to the pWOX5:BRI1-YFP; *bri1-116* roots could a dramatic reduction in meristem cell number be observed (Fig. S2A). This typical effect of exogenous BL application was not seen when just BRI1 is overexpressed. Together, these results suggest two possible scenarios: (1) there is an insufficient level of BRs in the root stem cell niche to promote QC division, or (2) BRI1-like receptors (i.e. BRL1 and BRL3) act as competitors for BR ligand binding.

To address the second scenario, we crossed the pWOX5:BRI1-YFP plants with double and triple mutants lacking two (*brl1brl3*) or all receptors (*bri1-116brl1brl3*), respectively, and assessed the occurrence of spontaneous QC divisions or an increased sensitivity to BL. Application of BL to the *brl1brl3* double mutant backgrounds yielded similar effects to those in the WT background, showing that the loss of these genes does not affect QC division rates even when applying lower concentrations of BL (0.04 nM) (Fig. S3, Table S2). With respect to the triple mutant, we obtained results similar to those found in the *bri1-116* background (Fig. S3, Table S2). Altogether, these results indicate that the BRL1/3 receptors do not compete with the BRI1 receptor for hormone binding. Interestingly, a lack of BRL receptors attenuates the slight increase in QC division that is observed upon overexpressing BRI1 in the QC (Fig. 2M; Fig. S3K, Table S2). In agreement with previously reported data (Fàbregas et al., 2013), this supports a marginal role for the BRL1and BRL3 receptors in promoting BR-mediated QC divisions in normal conditions. These results, together with the previous ones, exclude the possibility that BRL receptors compete with BRI1 for ligand binding. Thus, we conclude that the BR hormone concentration must be the limiting factor for promoting QC division.

### BRI1 is required in the stem cell niche for BL-triggered QC division

**Fig. 3.**
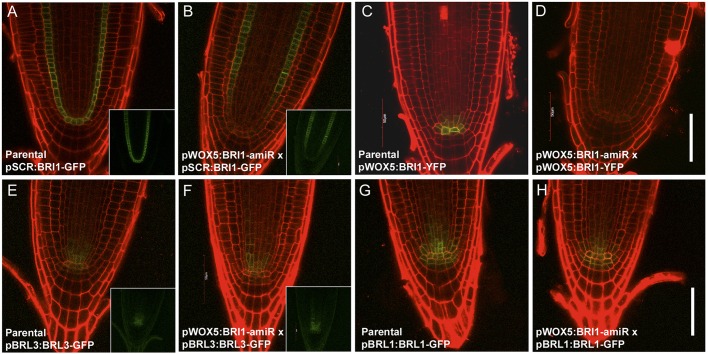
**The pWOX5:BRI1-amiR construct targets BRI1 and downregulates its transcription in the root stem cell microenvironment.** Confocal images of 6-day-old Arabidopsis roots. (A,B) Genetic crosses between pWOX5:BRI1-amiR and pSCR:BRI1-GFP lines reveal that BRI1 is knocked down in the stem cell microenvironment. (C,D) Genetic crosses between pWOX5:BRI1-YFP and pWOX5:BRI1-amiR lines show that the amiRNA completely depletes BRI1 around the QC domain. (E–H) Genetic crosses of pWOX5:BRI1-amiR lines with pBRL1:BRL1-GFP and pBRL3:BRL3-GFP lines. Insets show the GFP channel separately. All crosses are F3 double homozygous plants. Scale bar: 50 µm.

To more thoroughly understand the receptor requirements that drive BES1-mediated QC division, we specifically knocked out BRI1 expression in the WOX5 domain. For this, we designed and cloned an amiRNA against BRI1 (see Materials and Methods; Fig. S4A,B). To validate the ability of our amiRNA to knock out BRI1 expression, we first placed it under the control of the constitutive promoter CaMV35S. This resulted in dwarf plants similar to null *bri1* mutants (Li and Chory, 1997) (Fig. S4C). Next, cell-specific knockouts were generated by placing the amiRNA under the control of the QC-specific promoter *WOX5*. As seen by crossing pWOX5:BRI1-amiR plants with plants expressing BRI1-GFP under the control of the endodermis-specific promoter scarecrow (SCR) (Hacham et al., 2011), inhibition of BRI1 expression was not limited to the QC cells, but also occurred in nearby surrounding cells (Fig. 3A,B). This implies that the small size of the mature amiRNA enables it to diffuse to adjacent cells. Importantly, YFP signals observed in plants that overexpressed BRI1-YFP in the QC completely disappear when crossed with pWOX5:BRI1-amiR plants, indicating that our amiRNA is indeed effective at attenuating BRI1 expression (Fig. 3C,D). Finally, genetic crosses between the pWOX5:BRI1-amiR line and the translational reporter lines pBRL1:BRL1-GFP and pBRL3:BRL3-GFP (Fàbregas et al., 2013), showed that the BRI-amiR is partially depleting *BRL1* and *BRL3* transcripts, as consequence of sequence similarity (Fig. 3E–H). A GFP intensity reduction of ∼40% could be detected in the crosses (Fig. S5A,B).

**Fig. 4.**
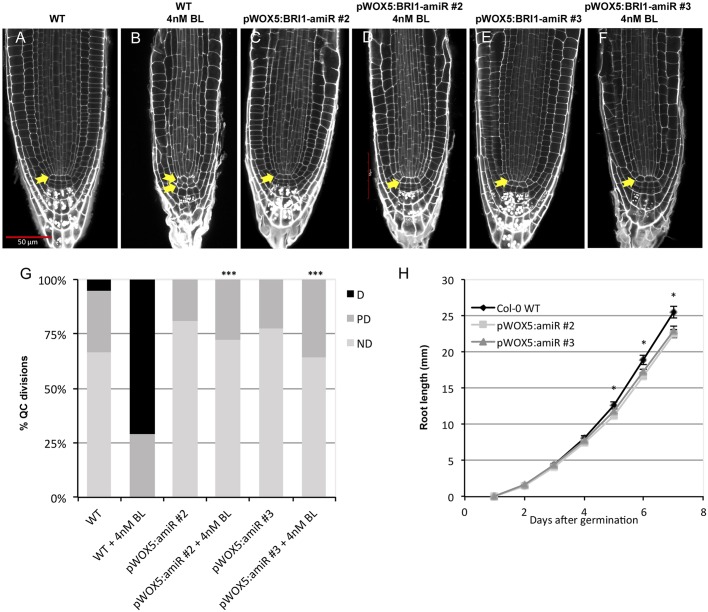
**BRI1 in the stem cells niche is required to promote QC divisions.** (A,B) Confocal images of 6-day-old WT Arabidopsis roots grown in either control conditions or 4 nM BL show the change in QC division and organization. (C–F) pWOX5:BRI1-amiR transgenic lines grown in control conditions or in medium supplemented with 4 nM BL. Arrows indicate the number of QC cell layers identified. (G) Quantification of the QC divisions of WT and pWOX5:BRI1-amiR plants. ND, QC non-divided; PD, QC partially divided; D, QC totally divided. Asterisks indicate statistically significant differences due to genotype, comparing against WT either in control or 4 nM BL conditions (***P<0.005). Frequencies in division occurrence were assessed with a two-sided Fisher's test. Values for all pairwise comparisons are provided in Table 3. Data generated from three independent replicates (*n*>39). (H) Root growth dynamics of WT and pWOX5:BRI1-amiR lines. Asterisks denote significant differences with respect to the WT in a two-tailed *t*-test (*P<0.05). Data are generated from three independent replicates (*n*>46). Scale bar: 50 µm.

Next, we analyzed two independent pWOX5:BRI1-amiR lines in terms of their sensitivity towards exogenous BL. Based on root length, meristem cell number and stele width, we found that both lines expressing the amiRNA retained a BL sensitivity closely similar to that of WT plants. In contrast, the null *bri1-116* plants were insensitive to hormone application (Fig. S2C–E), thereby suggesting that the effect of the mature amiRNA is strongly limited to a local level. Interestingly, both pWOX5:BRI1-amiR lines were completely insensitive to BL application in terms of QC division (Fig. 4A–G; Table S3). Taken together, these results indicate that the presence of BRI1 receptors in the QC is essential for QC division. Additionally, pWOX5:BRI1-amiR lines exhibited impaired root growth, having slightly, but significantly shorter roots than WT plants starting from 5 days after germination (Fig. 4H; Fig. S2C), suggesting that the presence of BR receptors in root stem cell niche contributes for optimal root growth.

**Fig. 5.**
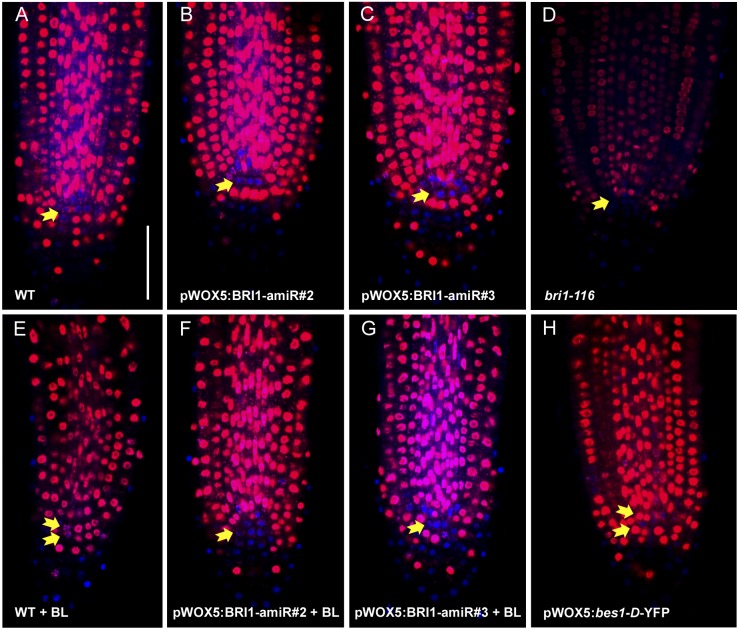
**pWOX5:BRI1-amiR seedlings exhibit normal meristem divisions.** Confocal images of fixed and EdU-stained 6-day-old Arabidopsis roots. (A–C) WT, pWOX5:BRI1-amiR#2 and pWOX5:BRI1-amiR#3 lines grown in control conditions. (D) *bri1-116* line grown in control conditions as a negative control for QC division. (E–G) WT, pWOX5:BRI1-amiR#2, and pWOX5:BRI1-amiR#3 lines grown for 4 days in control conditions and 2 days in medium supplemented with 4 nM BL. (H) pWOX5:*bes1-D*-YFP line grown in control conditions as a positive control for QC division. Arrows indicate the number of QC cell layers identified. Scale bar: 50 µm.

We next asked whether the reduction in QC divisions in the pWOX5:BRI1-amiR lines was a consequence of a slower cell cycle progression in the meristem. To answer this question, we stained roots with 5-ethynyl-2′-deoxyuridine (EdU), a thymidine analogue that is incorporated into actively dividing cells (Salic and Mitchison, 2008). In WT plants, we observed a uniform EdU staining in the entire root meristem except for in the QC, which owing to its quiescence, barely incorporates EdU (Fig. 5A). The same results, which are indicative of a normal cell cycle in the meristem, were also obtained for the pWOX5:BRI1-amiR lines (Fig. 5B,C). Thus, the QC remains quiescent because of the absence of BRI1, and not because of a meristem-wide deceleration of the cell cycle. In contrast, the *bri1-116* mutant showed a much lower extent of EdU incorporation, thereby confirming that it has a slower cell cycle compared with WT plants (Fig. 5D). Fluorescence intensity quantification confirmed that pWOX5:BRI1-amiR lines incorporate EdU at the same levels as in the WT, whereas *bri1-116* does so at lower rates (Fig. S5C) and it agrees with the previously reported slow cell cycle progression of *bri1-116* (Gonzalez-Garcia et al., 2011).

Furthermore, we treated both WT and pWOX5:BRI1-amiR lines with BL in order to evaluate whether BL promotes QC cell division. Upon BL treatment, WT roots incorporated EdU into the QC (Fig. 5E), thereby confirming that the QC cells were undergoing cell division. In contrast, however, the pWOX5:BRI1-amiR lines did not incorporate EdU into the QC after being subjected to identical BL treatment (Fig. 5F,G). This clearly supports the hypothesis that pWOX5:BRI1-amiR lines are insensitive to BR-mediated signals in the QC. Along the same lines, the plant that has a constitutively dividing QC due to overexpression of active BES1 (i.e. the pWOX5:*bes1-D*-YFP line), also exhibited EdU incorporation in the QC (Fig. 5H). This, in effect, mimics the results obtained with exogenous BL treatment, and confirms that activated downstream components of BR receptors are capable of triggering QC division in a cell-autonomous manner.

### Stem cell regeneration upon DNA damage entails the local action of BR receptors

**Fig. 6.**
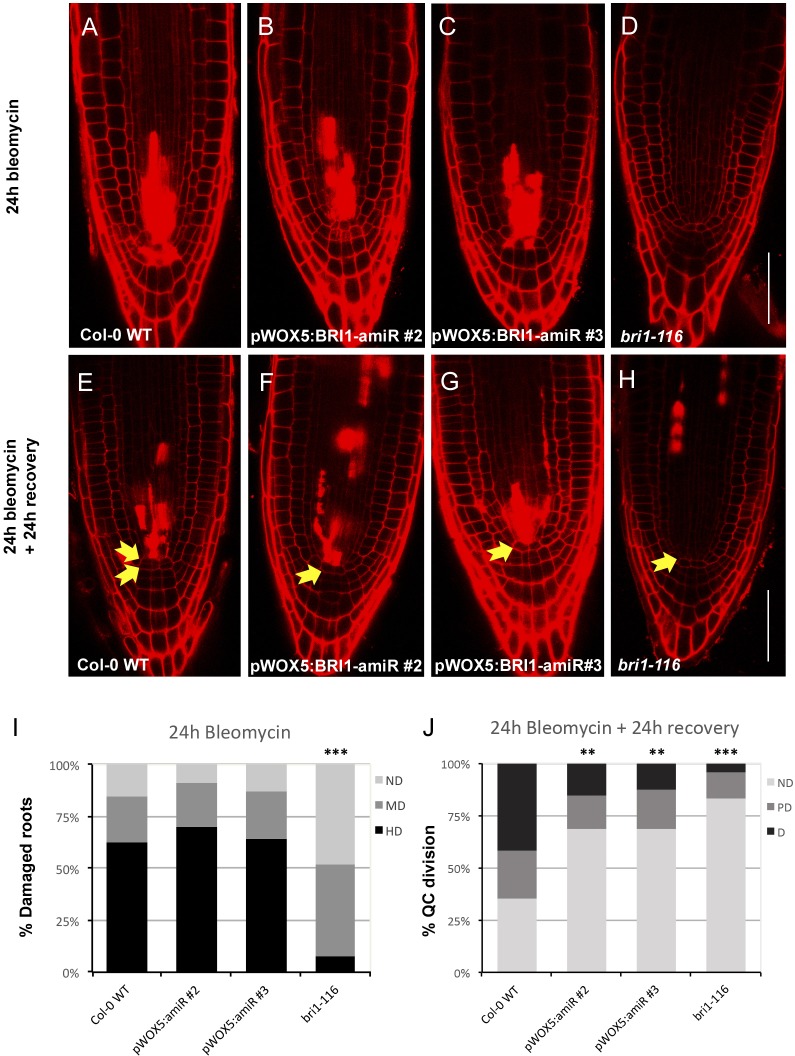
**BR receptors in the stem cell niche modulate QC divisions upon DNA damage.** (A–D) Confocal images of 5-day-old seedlings treated with bleomycin for 24 h. (E–H) Confocal images of 5-day-old seedlings subjected to 24 h of bleomycin treatment and a subsequent 24 h of recovery. (I) The proportion of roots showing cell death in the root apex after 24 h of bleomycin treatment. HD, hard damage; MD, mild damage; ND, no damage. Asterisks indicate statistically significant differences respect to WT (***P<0.005). Differences in the proportion of damaged roots were assessed with a two-sided Fisher's test. Values for all pairwise comparisons are provided in Table S4. Data are generated from three independent replicates (*n*>25). (J) Quantification of QC divisions after 24 h of bleomycin treatment and 24 additional hours of recovery. ND, QC non-divided; PD, QC partially divided; D, QC totally divided. Asterisks indicate statistically significant differences with respect to WT (**P<0.01, ***P<0.005). Differences in division frequencies were assessed with a two-sided Fisher's test. Values for all pairwise comparisons are provided in Table S5. Data are generated from three independent replicates (*n*>24). Scale bar: 50 µm.

Since the QC has been proposed to act as a stem cell reservoir and is known to divide in the face of environmental stresses, we decided to evaluate whether the BR receptors are essential for carrying out such stress-induced division. For this purpose, we decided to use bleomycin, a chemotherapeutic drug that has been described to preferentially harm root vascular stem cells and induce QC division (Fulcher and Sablowski, 2009; Vilarrasa-Blasi et al., 2014). As such, this system triggers QC division independently of BR treatment. We compared the local knockout lines (i.e. pWOX5:BRI1-amiR) against both the null *bri1* mutant and WT roots. While the pWOX5:BRI1-amiR lines were damaged at the same rate as the WT plants (Fig. 6A,B,C,I; Table S4), the *bri1* mutant remained free of any visible damage (Fig. 6D,I; Table S4). As previously described, this is probably due to its slow cell cycle progression (Gonzalez-Garcia et al., 2011; Vilarrasa-Blasi et al., 2014). Interestingly, in contrast to what was observed for the WT roots, the QC of the pWOX5:BRI1-amiR lines remained undivided following 24 h of bleomycin treatment plus 24 h of recovery (Fig. 6E,F,G,J; Table S5). In the case of *bri1*, the QC also remained undivided, but as previously mentioned, the roots were not damaged by bleomycin (Fig. 6H,J). Given that the pWOX5:BRI1-amiR lines and WT show similar levels of provascular cell death after 24 h of bleomycin treatment (Fig. 6A,B,C,I; Table S4), as well as the same amount of EdU staining (Fig. 5A–C; Fig. S5C), our results argue that the absence of QC divisions in bleomycin-treated pWOX5:BRI1-amiR lines is due to neither an inherent resistance against DNA damage nor a slow cell cycle progression. Interestingly, our results reveal the paracrine nature of this DNA damage response: a signal that emerges from damaged stem cells triggers cell division in the adjacent QC. Moreover, according to our data, this signal must be a type of steroid molecule that is locally and mainly transduced by BRI1 in the stem cell niche.

## DISCUSSION

The slow-dividing nature of the cells in the QC enable it to act as a cell reservoir and organizer for surrounding stem cells (Fulcher and Sablowski, 2009; Pi et al., 2015; Sarkar et al., 2007; van den Berg et al., 1997; Vilarrasa-Blasi et al., 2014). Although recent studies have started to shed light on the molecular components behind QC quiescence, the exact mechanisms that are responsible for ensuring such a low rate of cell division remain largely unknown. One fairly recent study discovered that the interaction between RETINOBLASTOME-RELATED (RBR) and SCARECROW (SCR) is required for quiescence maintenance (Cruz-Ramírez et al., 2013). Nonetheless, rather than being completely static, the QC is in fact regulated by plant hormone signaling. For instance, while it has been shown that abscisic acid (ABA) reinforces the quiescence of this group of cells (Zhang et al., 2010), ethylene (Ortega-Martinez et al., 2007) and cytokinin (Zhang et al., 2013) are known to disrupt their quiescence and promote division. With respect to BR hormones, they have been shown to promote QC divisions while maintaining regular cell cycle progression in the rest of the root meristem (Gonzalez-Garcia et al., 2011). The mechanisms underlying BR-mediated QC divisions are slowly being uncovered with the identification of BR-regulated and QC-specific transcription factors such as ERF115 (Heyman et al., 2013) and BRAVO (Vilarrasa-Blasi et al., 2014). However, how these signaling mechanisms are locally confined to the stem cell niche of the root is still controversial. In fact, although it has been proposed that BR action at the epidermis (Hacham et al., 2011) and vascular tissues (Kang et al., 2017) can similarly regulate meristem size and plant growth, it is unknown whether these local signals are also capable of driving QC divisions. Here, our findings show that QC activities at the stem cell niche require the presence of BR receptors in both the QC cells themselves and nearby surrounding cells.

### Activated BES1 can trigger cell-autonomous QC division but needs membrane support

Physiological analysis of QC-specific overexpression of BES1 revealed that active BES1 has the potential to trigger QC division in an autonomous manner. However, as the same QC division rates were not observed when the transgene was introduced into the *bri1* mutant background (Fig. 2M; Table S1), it became apparent that BRI1 was also required for this process. It is important to note that BRI1 might also activate other downstream components besides BES1. For example, one potential downstream target could be the transcription factor BZR1, which has been shown to promote autonomous QC division when activated (Chaiwanon and Wang, 2015; Lee et al., 2015). Interestingly, in the *bri1* background lines, we detected an increase in QC division frequency upon BL application (Fig. 2M; Table S1). This increase could be attributed to BRL receptors compensating for the lack of BRI1 and activating other downstream components.

### The hormone is the limiting factor for promoting QC divisions

Surprisingly, when the plants that overexpressed BRI1 in the QC (pWOX5:BRI1-YFP) were assessed in terms of QC division rates, we found only a limited increase in both the WT and *bri1* backgrounds (Fig. 2M; Table S1). The fact that the roots showed signs of recovery in the *bri1* background line (i.e. longer roots) however, confirmed that BRI1 was still functional when fused to YFP (Fig. S2B). Upon BL treatment, the QC division frequency of pWOX5:BRI1-YFP plants is similar to that in WT plants treated with BL (Fig. 2M; Table S1), thus revealing that an excess of receptor has no effect until the ligand is added. As the plants overexpressing pWOX5:BRI1-YFP displayed no dramatic phenotype until exogenous hormone was applied, we concluded that the stem cell niche microenvironment must be characterized by an excess of BRI1 and a limited amount of free hormone. We discounted competition for the ligand between BRI1 and BRLs as the reason for this (Fig. S3, Table S2), and hypothesize that, in the root stem cell niche, a threshold of available hormone has to be reached in order to promote QC divisions.

### BRI1 is necessary but not sufficient to promote QC division

According to our results, the presence of BRI1 in the QC is not the limiting factor for the QC division process. In fact, very low amounts of BRI1 receptor are present within these cells (Wilma van Esse et al., 2011). Furthermore, BRL1 and BRL3, both of which bind the hormone with a higher affinity than BRI1, are also present in these cells (Cano-Delgado et al., 2004; Fàbregas et al., 2013). Accordingly, we wondered whether BRI1 was absolutely necessary in this domain. Our results show that WT lines expressing the amiRNA against BRI1 in the stem cell niche (pWOX5:BRI1-amiR) are completely insensitive towards BL-induced QC divisions (Fig. 4E). At the same time, however, BRI1 acting exclusively in the QC (i.e. pWOX5:BRI1-YFP; *bri1-116* line) is not enough to recover BL-induced QC divisions to WT levels (Fig. 2M). Taken together, these results suggest that the effects of BRI1 are reinforced from surrounding cells. Thus, we found that BRI1 signaling in the QC is necessary, but not sufficient to promote QC self-renewal, and highlight BRI1 as the main driving factor for this process. Despite the fact that BRL activity is also partially downregulated in pWOX5:BRI1-amiR lines, in agreement with our data, previous results showed that *brl1brl3* double mutants have a normal BR-induced QC division (Fábregas et al., 2013). On the other hand, *bri1-116* mutants, which have intact *BRL1* and *BRL3* genes, retain a quiescent QC, even upon application of high doses of BL (Gonzalez-Garcia et al., 2011) (Fig. 2M; Table S1). Our results relegate BRL receptors to a supporting action for BRI1, which in turn acts as the main promoter of QC divisions in normal conditions. Moreover, QC division frequency also has an impact on the growth of primary roots, as the roots of pWOX5:BRI1-amiR lines are slightly shorter than those of the WT (Fig. 4H; Fig. S2C). Congruently, the *bri1-116* mutant lines that overexpressed BRI1 or BES1 in the QC (i.e. pWOX5:BRI1-YFP;*bri1-116* and pWOX5:bes1-D-YFP;bri1-116) not only partially recovered BR signaling in the QC, but also partially recovered seedling root length compared with that in the *bri1-116* mutant (Fig. S2D). This latter fact prompted us to hypothesize that some spontaneous QC divisions under basal conditions are required to sustain optimal root growth – presumably for replenishment of the stem cell niche.

### BR signaling acts in a paracrine manner to trigger QC division

It is known that the QC divides in response to environmental stresses such as the presence of DNA-damaging agents (Vilarrasa-Blasi et al., 2014) or changes in the homeostasis of reactive oxygen species (ROS) (Yu et al., 2016). In the root, DNA-damaging agents preferentially harm vascular and columella stem cells. Cells that are unable to repair this damage activate programmed cell death (PCD) and undergo apoptosis (Fulcher and Sablowski, 2009), thereby subsequently promoting QC divisions to replenish the stem cell niche and maintain meristematic activities (Heyman et al., 2016; Vilarrasa-Blasi et al., 2014). We took advantage of this property to analyze the receptor requirements of the signaling that causes QC division. Interestingly, we found that the BRI1 receptor is necessary to trigger QC divisions after vascular cell death (Fig. 6), although we cannot discard a major contribution of BRLs under this stress scenario. Furthermore, we discounted the idea that QC quiescence observed in the pWOX5:BRI1-amiR line after damage is due to a slower cell cycle (Fig. 5; Fig. S5C), as is the case for the *bri1-116* mutant. Although it has been demonstrated that downregulation of BRAVO is implicated in this type of QC division (Vilarrasa-Blasi et al., 2014), the exact nature of signal progression from the damaged cell to the QC is still unclear. Even if we cannot discern between BRI1 and the BRLs perceiving this signal, results obtained by treating the pWOX5:BRI1-amiR lines with bleomycin have revealed that these signals are perceived by BR receptors acting in the stem cell niche, so the signal should be of a steroid nature and act in a paracrine manner.

It is known that by stimulating paracrine signaling, human stem cells can promote wound healing and cancer progression (Dittmer and Leyh, 2014), but in plants, the mechanisms behind autocrine and paracrine signaling are only just being uncovered (Qi et al., 2017). It has been proposed that BRs can regulate stem cell division in the roots via long-range signals originating at the epidermis (Hacham et al., 2011). However, although changes in QC markers (e.g. AGL42) were observed in response to epidermal signaling, no effect on QC divisions was reported (Hacham et al., 2011). This therefore limits direct readout of BR-mediated signaling in the QC to short-range signals. Indeed, in contrast to other hormones that act over long distances, it is accepted that BRs act at a more local level (Fridman et al., 2014) and our findings indicate that the signals that promote QC divisions come from the nearby stem cell microenvironment rather than from the outer cell layers. Nevertheless, where exactly the BR signals are driven from remains a controversy.

**Fig. 7.**
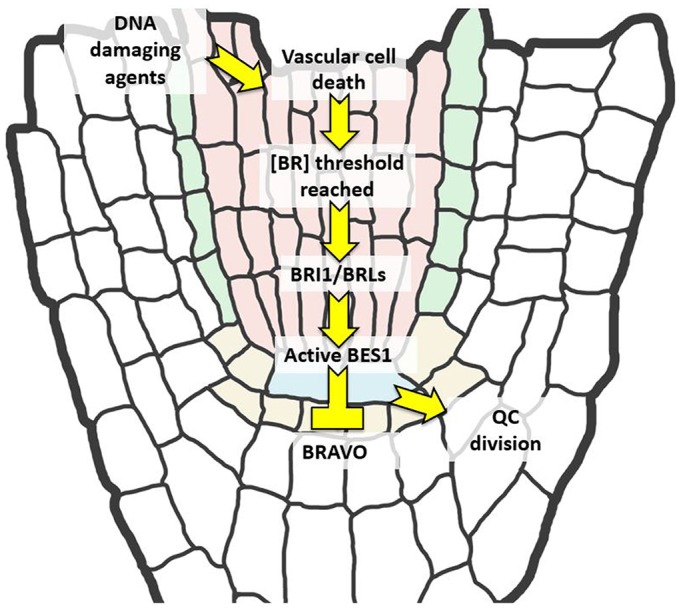
**Working model: BR concentration as a limiting factor for QC divisions.** In order to promote QC divisions when needed, a threshold concentration of BRs has to be reached in the root apical meristem. Upon reaching this threshold, the signal is transduced via BRI1 with enough strength to promote BES1 dephosphorylation. Dephosphorylated BES1, in turn, inhibits BRAVO and triggers QC division.

In summary, our findings show that (1) QC cell division activity is promoted by BES1 transcription factor in the QC; (2) BRI1 is required in both the QC and nearby cells to trigger division; and (3) paracrine steroid signaling may be regulated by the hormone's availability in the stem cell niche (Fig. 7). A plausible way to control the hormone levels in the stem cell microenvironment of the root could be to upregulate the genes controlling its biosynthesis. However, the spatial regulation of the enzymes responsible for BR biosynthesis is still poorly understood. As such, further efforts in this area are crucial for elucidating the nature and origin of BR signals, where they are synthesized and where they are driven.

## MATERIAL AND METHODS

### Plant material and growth conditions

All lines used in this study, along with their references are listed in Table S6. We used *Arabidopsis thaliana* (L.) Heyhn, ecotype Columbia-0 (Col-0) as the control background line.

Seeds were surface sterilized using 35% bleach, and subsequently washed five times with distilled sterile water. Seeds were vernalized at 4°C in the dark for 48 h before sowing. Plants were grown in vertical plates containing half-strength Murashige and Skoog (MS) medium with vitamins but no sucrose supplements (0.5×MS−), in long day conditions (LD, 16 h light:8 h dark) at 22°C and 60% relative humidity.

### amiRNA design and cloning

We designed the artificial miRNA using Web MicroRNA Designer (WMD2) as previously described (Ossowski et al., 2008; Schwab et al., 2006). Briefly, the nucleotides encoding the mature miRNA sequence, GCCCCTATCTAAGTGTCAGTT, were engineered in the miR319a precursor as described (Schwab et al., 2006). This was then subcloned under the control of the WOX5 QC promoter in the binary plasmid pH7m24GW,3, and transformed into Arabidopsis using the floral dip method (Zhang et al., 2006). In this work, we used two independent homozygous T4 lines named pWOX5:BRI1-amiR#2 and pWOX5:BRI1-amiR#3, both of which express the specific amiRNA against BRI1 under the *WOX5* promoter (4.2 kb upstream of the *WOX5* start codon). For the pWOX5:BRI1-YFP construct, the coding sequence of the *BRI1* gene was cloned under the control of the *WOX5* promoter and fused to *YFP*, all inside the binary plasmid pB7m34GW. All constructs were cloned using Gateway technology (Invitrogen) according to the manufacturer's instructions.

### Confocal microscopy

For QC division analysis, 6-day-old seedlings were fixed, clarified and counterstained using modified Pseudo Schiff–propidium iodide (mPS-PI) staining (Truernit and Haseloff, 2008). Then, each seedling was mounted onto a microscope slide with a drop of Hoyer's solution (30 g gum arabic, 200 g chloral hydrate, 20 g glycerol and 50 ml water). Images were obtained using a FV 1000 confocal microscope (Olympus, Tokyo, Japan). The QC division phenotypes were scored as in Vilarrasa-Blasi et al. (2014). Differences in QC division frequencies were statistically evaluated with a two-sided Fisher's exact test (Tables S2–S4).

For bleomycin assays, the percentage of damaged roots was scored after 24 h of treatment, which is a qualitative classification depending on the amount of death cells in the vasculature, identified by the incorporation of PI inside the cells: no damage means that cells did not uptake PI; mid damage indicates that some cells in the stem cell niche area were stained; hard damage indicates that all cells in the stem cell niche and some cells in the vascular system stained with PI. The percentage of QC divisions was scored after 24 h of bleomycin treatment and 24 h of recovery.

### Hormone and drug treatments

For brassinolide (BL) treatment, BL (C28H48O6; Wako, Osaka, Japan) previously dissolved in ethanol was added to medium at a final concentration of either 4 nM or 0.04 nM. For bleomycin treatment, seedlings were transferred to vertical plates supplemented with 0.6 µg/ml bleomycin (Calbiochem) 4 days after sowing. For recovery, plants were transferred back to control medium after 1 day of growth in bleomycin-containing medium and quantified under a confocal microscope after 24 h.

### EdU staining

For evaluating EdU staining, we used the Click-iT EdU Alexa Fluor 555 Imaging Kit (Thermo Fisher). Five days after sowing, seedlings were transferred to vertical plates supplemented with 10 µg/ml EdU. After 24 h, seedlings were fixed in a solution containing 3.7% (w/v) paraformaldehyde and 1% (v/v) Triton X-100 in 1× PBS for 1 h in a vacuum. After fixation, the seedlings were washed twice with 3% (w/v) BSA in 1× PBS, and subsequently incubated in the Click-iT reaction cocktail (as described in the protocol of Invitrogen EdU Click-iT Reaction Imaging Kit) for 1 h in the dark. For counterstaining, seedlings were washed twice with 3% BSA in 1× PBS and incubated for 30 min with 1 µg/ml DAPI in 1× PBS in the dark. Finally, the seedlings were washed a final time in 3% BSA in 1× PBS.

### Root measurements and fluorescence quantification

For root length measurements, images of seedlings were taken with a Nikon D7000 camera and roots were measured with ImageJ software (http://imagej.nih.gov/ij/). For meristem cell counts, 6-day-old seedlings were stained with 10 µg/ml PI and the images were obtained using a FV 1000 confocal microscope (Olympus, Tokyo, Japan), using a 20× objective. Then cells were counted by tracking the cortex, starting from QC cells. The end of the meristem was considered when a cell had >75% increase in cell length (longitudinally) than the previous one. Cell measurements were performed with ImageJ. For root stele width, measures were taken at 50 µm upstream of the QC in the root longitudinal axis. The separation between pericycle cell files (stele) was measured perpendicular to the root longitudinal axis. Measures were made with ImageJ. For fluorescence quantifications, the mean pixels/area of fluorescence in the green channel (to quantify GFP) or the red channel (to quantify EdU incorporation) were quantified with ImageJ, either on complete images for the EdU-stained samples or by measuring only the area of expression of the BRLs.

## Supplementary Material

Supplementary information

## References

[JCS204065C1] Aranda A. and Pascual A. (2001). Nuclear hormone receptors and gene expression. *Physiol. Rev.* 81, 1269 10.1152/physrev.2001.81.3.1269 11427696

[JCS204065C2] Cano-Delgado A., Yin Y., Yu C., Vafeados D., Mora-Garcia S., Cheng J. C., Nam K. H., Li J. and Chory J. (2004). BRL1 and BRL3 are novel brassinosteroid receptors that function in vascular differentiation in Arabidopsis. *Development* 131, 5341-5351. 10.1242/dev.01403 15486337

[JCS204065C3] Chaiwanon J. and Wang Z.-Y. (2015). Spatiotemporal brassinosteroid signaling and antagonism with auxin pattern stem cell dynamics in Arabidopsis roots. *Curr. Biol.* 25, 1031-1042. 10.1016/j.cub.2015.02.046 25866388 PMC4415608

[JCS204065C4] Cruz-Ramírez A., Díaz-Triviño S., Wachsman G., Du Y., Arteága-Vázquez M., Zhang H., Benjamins R., Blilou I., Neef A. B., Chandler V. et al. (2013). A SCARECROW-RETINOBLASTOMA protein network controls protective quiescence in the Arabidopsis root stem cell organizer. *PLoS Biol.* 11, e1001724 10.1371/journal.pbio.1001724 24302889 PMC3841101

[JCS204065C5] Dittmer J. and Leyh B. (2014). Paracrine effects of stem cells in wound healing and cancer progression (Review). *Int. J. Oncol.* 44, 1789-1798. 10.3892/ijo.2014.2385 24728412 PMC4063537

[JCS204065C6] Dolan L., Janmaat K., Willemsen V., Linstead P., Poethig S., Roberts K. and Scheres B. (1993). Cellular organisation of the Arabidopsis thaliana root. *Development* 119, 71-84. 8275865 10.1242/dev.119.1.71

[JCS204065C7] Fàbregas N., Li N., Boeren S., Nash T. E., Goshe M. B., Clouse S. D., de Vries S. and Cano-Delgado A. I. (2013). The brassinosteroid insensitive1-like3 signalosome complex regulates Arabidopsis root development. *Plant Cell* 25, 3377-3388. 10.1105/tpc.113.114462 24064770 PMC3809538

[JCS204065C8] Fridman Y., Elkouby L., Holland N., Vragovic K., Elbaum R. and Savaldi-Goldstein S. (2014). Root growth is modulated by differential hormonal sensitivity in neighboring cells. *Genes Dev.* 28, 912-920. 10.1101/gad.239335.114 24736847 PMC4003282

[JCS204065C9] Friedrichsen D. and Chory J. (2001). Steroid signaling in plants: from the cell surface to the nucleus. *BioEssays* 23, 1028-1036. 10.1002/bies.1148 11746219

[JCS204065C10] Fulcher N. and Sablowski R. (2009). Hypersensitivity to DNA damage in plant stem cell niches. *Proc. Natl Acad. Sci. USA* 106, 20984-20988. 10.1073/pnas.0909218106 19933334 PMC2791609

[JCS204065C11] Geldner N., Hyman D. L., Wang X., Schumacher K. and Chory J. (2007). Endosomal signaling of plant steroid receptor kinase BRI1. *Genes Dev.* 21, 1598-1602. 10.1101/gad.1561307 17578906 PMC1899468

[JCS204065C12] Gonzalez-Garcia M.-P., Vilarrasa-Blasi J., Zhiponova M., Divol F., Mora-Garcia S., Russinova E. and Cano-Delgado A. I. (2011). Brassinosteroids control meristem size by promoting cell cycle progression in Arabidopsis roots. *Development* 138, 849-859. 10.1242/dev.057331 21270057

[JCS204065C13] Hacham Y., Holland N., Butterfield C., Ubeda-Tomas S., Bennett M. J., Chory J. and Savaldi-Goldstein S. (2011). Brassinosteroid perception in the epidermis controls root meristem size. *Development* 138, 839-848. 10.1242/dev.061804 21270053 PMC3035089

[JCS204065C14] He J.-X., Gendron J. M., Yang Y., Li J. and Wang Z.-Y. (2002). The GSK3-like kinase BIN2 phosphorylates and destabilizes BZR1, a positive regulator of the brassinosteroid signaling pathway in Arabidopsis. *Proc. Natl. Acad. Sci. USA* 99, 10185-10190. 10.1073/pnas.152342599 12114546 PMC126645

[JCS204065C15] He J.-X., Gendron J. M., Sun Y., Gampala S. S., Gendron N., Sun C. Q. and Wang Z. Y. (2005). BZR1 is a transcriptional repressor with dual roles in brassinosteroid homeostasis and growth responses. *Science* 307, 1634-1638. 10.1126/science.1107580 15681342 PMC2925132

[JCS204065C16] Heyman J., Cools T., Vandenbussche F., Heyndrickx K. S., Van Leene J., Vercauteren I., Vanderauwera S., Vandepoele K., De Jaeger G., Van Der Straeten D. et al. (2013). ERF115 controls root quiescent center cell division and stem cell replenishment. *Science* 342, 860-863. 10.1126/science.1240667 24158907

[JCS204065C17] Heyman J., Cools T., Canher B., Shavialenka S., Traas J., Vercauteren I., Van, den Daele H., Persiau G., De Jaeger G. et al. (2016). The heterodimeric transcription factor complex ERF115-PAT1 grants regeneration competence. *Nat. Plants* 2, 16165 10.1038/nplants.2016.165 27797356

[JCS204065C18] Hothorn M., Belkhadir Y., Dreux M., Dabi T., Noel J. P., Wilson I. A. and Chory J. (2011). Structural basis of steroid hormone perception by the receptor kinase BRI1. *Nature* 474, 467-471. 10.1038/nature10153 21666665 PMC3280218

[JCS204065C19] Kang Y. H., Breda A. and Hardtke C. S. (2017). Brassinosteroid signaling directs formative cell divisions and protophloem differentiation in Arabidopsis root meristems. *Development* 144, 272-280. 10.1242/dev.145623 28096215 PMC5394764

[JCS204065C20] Kim T.-W. and Wang Z.-Y. (2010). Brassinosteroid signal transduction from receptor kinases to transcription factors. *Annu. Rev. Plant Biol.* 61, 681-704. 10.1146/annurev.arplant.043008.092057 20192752

[JCS204065C21] Kim B., Jeong Y. J., Corvalán C., Fujioka S., Cho S., Park T. and Choe S. (2014). Darkness and gulliver2/phyB mutation decrease the abundance of phosphorylated BZR1 to activate brassinosteroid signaling in Arabidopsis. *Plant J.* 77, 737-747. 10.1111/tpj.12423 24387668 PMC4282538

[JCS204065C22] Kinoshita T., Caño-Delgado A., Seto H., Hiranuma S., Fujioka S., Yoshida S. and Chory J. (2005). Binding of brassinosteroids to the extracellular domain of plant receptor kinase BRI1. *Nature* 433, 167-171. 10.1038/nature03227 15650741

[JCS204065C23] Lee H.-S., Kim Y., Pham G., Kim J. W., Song J.-H., Lee Y., Hwang Y.-S., Roux S. J. and Kim S.-H. (2015). Brassinazole resistant 1 (BZR1)-dependent brassinosteroid signalling pathway leads to ectopic activation of quiescent cell division and suppresses columella stem cell differentiation. *J. Exp. Bot.* 66, 4835-4849. 10.1093/jxb/erv316 26136267 PMC4507784

[JCS204065C24] Li J. and Chory J. (1997). A putative leucine-rich repeat receptor kinase involved in brassinosteroid signal transduction. *Cell* 90, 929-938. 10.1016/S0092-8674(00)80357-8 9298904

[JCS204065C25] Li J. and Nam K. H. (2002). Regulation of brassinosteroid signaling by a GSK3/SHAGGY-like kinase. *Science* 295, 1299-1301. 10.1126/science.1065769 11847343

[JCS204065C26] Mitchell J. W., Mandava N., Worley J. F., Plimmer J. R. and Smith M. V. (1970). Brassins–a new family of plant hormones from rape pollen. *Nature* 225, 1065-1066. 10.1038/2251065a0 16056912

[JCS204065C27] Nolan T. M., Brennan B., Yang M., Chen J., Zhang M., Li Z., Wang X., Bassham D. C., Walley J. and Yin Y. (2017). Selective autophagy of BES1 mediated by DSK2 balances plant growth and survival. *Dev. Cell* 41, 33-46.e37. 10.1016/j.devcel.2017.03.013 28399398 PMC5720862

[JCS204065C28] Ortega-Martinez O., Pernas M., Carol R. J. and Dolan L. (2007). Ethylene modulates stem cell division in the Arabidopsis thaliana root. *Science* 317, 507-510. 10.1126/science.1143409 17656722

[JCS204065C29] Ossowski S., Schwab R. and Weigel D. (2008). Gene silencing in plants using artificial microRNAs and other small RNAs. *Plant J.* 53, 674-690. 10.1111/j.1365-313X.2007.03328.x 18269576

[JCS204065C30] Peng P., Yan Z., Zhu Y. and Li J. (2008). Regulation of the Arabidopsis GSK3-like kinase BRASSINOSTEROID-INSENSITIVE 2 through proteasome-mediated protein degradation. *Mol. Plant* 1, 338-346. 10.1093/mp/ssn001 18726001 PMC2519614

[JCS204065C31] Pi L., Aichinger E., van der Graaff E., Llavata-Peris C. I., Weijers D., Hennig L., Groot E. and Laux T. (2015). Organizer-Derived WOX5 signal maintains root columella stem cells through chromatin-mediated repression of CDF4 expression. *Dev. Cell*. 33, 576-588. 10.1016/j.devcel.2015.04.024 26028217

[JCS204065C32] Qi X., Han S. K., Dang J. H., Garrick J. M., Ito M., Hofstetter A. K. and Torii K. U. (2017). Autocrine regulation of stomatal differentiation potential by EPF1 and ERECTA-LIKE1 ligand-receptor signaling. *Elife* 6, e24102 10.7554/eLife.24102 28266915 PMC5358980

[JCS204065C33] Russinova E., Borst J.-W., Kwaaitaal M., Caño-Delgado A., Yin Y., Chory J. and de Vries S. C. (2004). Heterodimerization and endocytosis of Arabidopsis brassinosteroid receptors BRI1 and AtSERK3 (BAK1). *Plant Cell* 16, 3216-3229. 10.1105/tpc.104.025387 15548744 PMC535869

[JCS204065C34] Sabatini S., Heidstra R., Wildwater M. and Scheres B. (2003). SCARECROW is involved in positioning the stem cell niche in the Arabidopsis root meristem. *Genes Dev.* 17, 354-358. 10.1101/gad.252503 12569126 PMC195985

[JCS204065C35] Sablowski R. (2004). Plant and animal stem cells: conceptually similar, molecularly distinct? *Trends Cell Biol.* 14, 605-611. 10.1016/j.tcb.2004.09.011 15519849

[JCS204065C36] Salazar-Henao J. E., Lehner R., Betegón-Putze I., Vilarrasa-Blasi J. and Caño-Delgado A. I. (2016). BES1 regulates the localization of the brassinosteroid receptor BRL3 within the provascular tissue of the Arabidopsis primary root. *J. Exp. Bot.* 67, 4951-4961. 10.1093/jxb/erw258 27511026 PMC5014150

[JCS204065C37] Salic A. and Mitchison T. J. (2008). A chemical method for fast and sensitive detection of DNA synthesis in vivo. *Proc. Natl. Acad. Sci. USA* 105, 2415-2420. 10.1073/pnas.0712168105 18272492 PMC2268151

[JCS204065C38] Sarkar A. K., Luijten M., Miyashima S., Lenhard M., Hashimoto T., Nakajima K., Scheres B., Heidstra R. and Laux T. (2007). Conserved factors regulate signalling in Arabidopsis thaliana shoot and root stem cell organizers. *Nature* 446, 811-814. 10.1038/nature05703 17429400

[JCS204065C55] Scheres B. (2007). Stem-cell niches: nursery rhymes across kingdoms. *Nature Rev. Mol. Cell Biol.* 8, 345 10.1038/nrm2164 17450175

[JCS204065C39] Schwab R., Ossowski S., Riester M., Warthmann N. and Weigel D. (2006). Highly specific gene silencing by artificial microRNAs in arabidopsis. *Plant Cell* 18, 1121-1133. 10.1105/tpc.105.039834 16531494 PMC1456875

[JCS204065C40] Sun Y., Fan X.-Y., Cao D.-M., Tang W., He K., Zhu J.-Y., He J.-X., Bai M.-Y., Zhu S., Oh E. et al. (2010). Integration of brassinosteroid signal transduction with the transcription network for plant growth regulation in Arabidopsis. *Dev. Cell* 19, 765-777. 10.1016/j.devcel.2010.10.010 21074725 PMC3018842

[JCS204065C41] Thummel C. S. and Chory J. (2002). Steroid signaling in plants and insects–common themes, different pathways. *Genes Dev.* 16, 3113-3129. 10.1101/gad.1042102 12502734

[JCS204065C42] Truernit E. and Haseloff J. (2008). A simple way to identify non-viable cells within living plant tissue using confocal microscopy. *Plant Methods* 4, 15 10.1186/1746-4811-4-15 18573203 PMC2442066

[JCS204065C43] van den Berg C., Willemsen V., Hendriks G., Weisbeek P. and Scheres B. (1997). Short-range control of cell differentiation in the Arabidopsis root meristem. *Nature* 390, 287-289. 10.1038/36856 9384380

[JCS204065C44] Vilarrasa-Blasi J., González-García M.-P., Frigola D., Fàbregas N., Alexiou K. G., López-Bigas N., Rivas S., Jauneau A., Lohmann J. U., Benfey P. N. et al. (2014). Regulation of plant stem cell quiescence by a brassinosteroid signaling module. *Dev. Cell* 30, 36-47. 10.1016/j.devcel.2014.05.020 24981610

[JCS204065C45] Wang Z.-Y., Seto H., Fujioka S., Yoshida S. and Chory J. (2001). BRI1 is a critical component of a plasma-membrane receptor for plant steroids. *Nature* 410, 380-383. 10.1038/35066597 11268216

[JCS204065C46] Wang Z.-Y., Nakano T., Gendron J., He J., Chen M., Vafeados D., Yang Y., Fujioka S., Yoshida S., Asami T. et al. (2002). Nuclear-localized BZR1 mediates brassinosteroid-induced growth and feedback suppression of brassinosteroid biosynthesis. *Dev. Cell* 2, 505-513. 10.1016/S1534-5807(02)00153-3 11970900

[JCS204065C47] Wei Z. and Li J. (2016). Brassinosteroids regulate root growth, development, and symbiosis. *Mol. Plant* 9, 86-100. 10.1016/j.molp.2015.12.003 26700030

[JCS204065C48] Wilma van Esse G., Westphal A. H., Surendran R. P., Albrecht C., van Veen B., Borst J. W. and de Vries S. C. (2011). Quantification of the brassinosteroid insensitive1 receptor in planta. *Plant Physiol.* 156, 1691 10.1104/pp.111.179309 21617031 PMC3149942

[JCS204065C49] Yin Y., Wang Z.-Y., Mora-Garcia S., Li J., Yoshida S., Asami T. and Chory J. (2002). BES1 accumulates in the nucleus in response to brassinosteroids to regulate gene expression and promote stem elongation. *Cell* 109, 181-191. 10.1016/S0092-8674(02)00721-3 12007405

[JCS204065C50] Yu X., Li L., Zola J., Aluru M., Ye H., Foudree A., Guo H., Anderson S., Aluru S., Liu P. et al. (2011). A brassinosteroid transcriptional network revealed by genome-wide identification of BESI target genes in Arabidopsis thaliana. *Plant J.* 65, 634-646. 10.1111/j.1365-313X.2010.04449.x 21214652

[JCS204065C51] Yu Q., Tian H., Yue K., Liu J., Zhang B., Li X. and Ding Z. (2016). A P-loop NTPase regulates quiescent center cell division and distal stem cell identity through the regulation of ROS homeostasis in arabidopsis root. *PLoS Genet.* 12, e1006175 10.1371/journal.pgen.1006175 27583367 PMC5008728

[JCS204065C52] Zhang X., Henriques R., Lin S.-S., Niu Q.-W. and Chua N.-H. (2006). Agrobacterium-mediated transformation of Arabidopsis thaliana using the floral dip method. *Nat. Protoc.* 1, 641-646. 10.1038/nprot.2006.97 17406292

[JCS204065C53] Zhang H., Han W., De Smet I., Talboys P., Loya R., Hassan A., Rong H., Jürgens G., Paul Knox J. and Wang M.-H. (2010). ABA promotes quiescence of the quiescent centre and suppresses stem cell differentiation in the Arabidopsis primary root meristem. *Plant J.* 64, 764-774. 10.1111/j.1365-313X.2010.04367.x 21105924

[JCS204065C54] Zhang W., Swarup R., Bennett M., Schaller G. E. and Kieber J. J. (2013). Cytokinin induces cell division in the quiescent center of the arabidopsis root apical meristem. *Curr. Biol.* 23, 1979-1989. 10.1016/j.cub.2013.08.008 24120642

