# Supplementary material for: Paracrine brassinosteroid signaling at the stem cell niche controls cellular regeneration
Source: J Cell Sci. 2018 Jan 15;131(2):jcs204065. doi: 10.1242/jcs.204065 (PMC5818034; doi:10.1242/jcs.204065)
Supplement: Supplementary information [file joces-131-204065-s1.pdf]

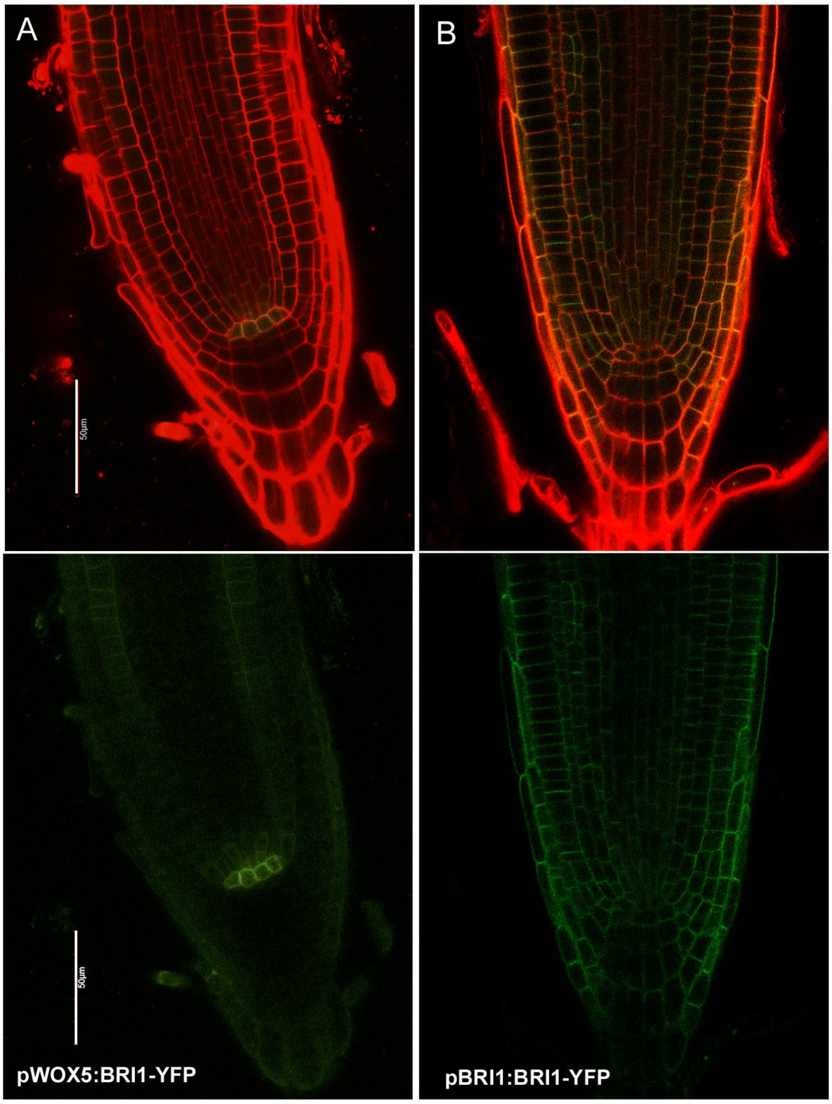

Figure S1. WOX5-controlled BRI1 expression is QC-specific.

A-B) Confocal images of 6-day-old Arabidopsis roots grown under control conditions. pWOX5:BRI1-YFP (A) and pBRI1:BRI1-GFP (B) (Geldner et al., 2007). Green represents the GFP or YFP-tagged BRI1protein while red is the PI counterstain. Scale bar: 50 µm.

Journal of Cell Science • Supplementary information

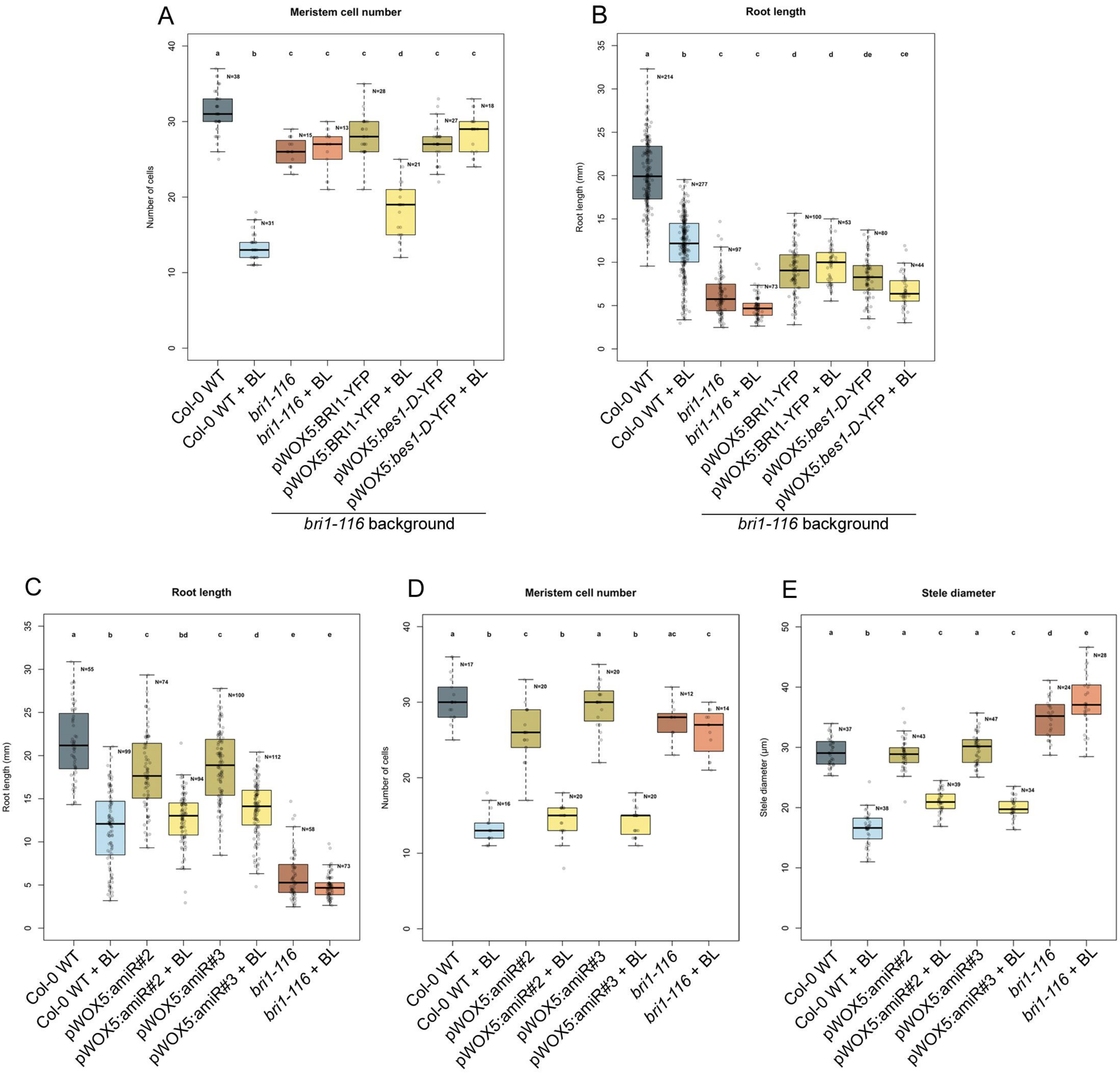
A
Meristem cell number
Number of cells
Col-0 WT
Col-0 WT + BL
bri1-116
bri1-116 + BL
pWOX5:BRI1-YFP
pWOX5:BRI1-YFP + BL
pWOX5:bes1-D-YFP
pWOX5:bes1-D-YFP + BL
bri1-116 background
B
Root length
Root length (mm)
Col-0 WT
Col-0 WT + BL
bri1-116
bri1-116 + BL
pWOX5:BRI1-YFP
pWOX5:BRI1-YFP + BL
pWOX5:bes1-D-YFP
pWOX5:bes1-D-YFP + BL
bri1-116 background
C
Root length
Root length (mm)
Col-0 WT
Col-0 WT + BL
pWOX5:amiR#2
pWOX5:amiR#2 + BL
pWOX5:amiR#3
pWOX5:amiR#3 + BL
bri1-116
bri1-116 + BL
D
Meristem cell number
Number of cells
Col-0 WT
Col-0 WT + BL
pWOX5:amiR#2
pWOX5:amiR#2 + BL
pWOX5:amiR#3
pWOX5:amiR#3 + BL
bri1-116
bri1-116 + BL
E
Stele diameter
Stele diameter (µm)
Col-0 WT
Col-0 WT + BL
pWOX5:amiR#2
pWOX5:amiR#2 + BL
pWOX5:amiR#3
pWOX5:amiR#3 + BL
bri1-116
bri1-116 + BL

Figure S2. QC-specific expression of BR components has an impact on the growth of primary roots, and pWOX5:BRI1-amiR lines retain sensitivity to BL.

A) Quantification of meristem cell number of QC-overexpression lines upon BL application. Expressing BRI1 exclusively in the QC partially recovers the sensitivity to BL application and thus showing BRI1 is active in the WOX5 domain. **B)** Quantification of root length of QC-overexpression lines. The partial alleviation of *bri1-116* dwarf phenotype in pWOX5:BRI1-YFP lines suggests that BR signaling in the QC accounts for overall root growth. **C)** Quantification of root length of pWOX5:BRI1-amiR lines shows the depletion of BRI1 in QC and surrounding cells negatively affects overall root growth. **D)** Quantification of meristem cell number of pWOX5:BRI1-amiR lines shows that seedlings retain sensitivity to exogenous BL applications. **E)** Quantification of stele width of pWOX5:BRI1-amiR lines. Data shows that pWOX5:BRI1-amiR lines do not have affected stele width. However, they are slightly less sensitive to BL applications. Interestingly, *bri1-116* null mutant has a wider stele, which is even more expanded upon BL application. All pairwise comparisons were evaluated trough one-way ANOVA and Tukey post-hoc test. Different letters above the boxplots mean statistically significant differences. Data are generated from three independent replicates. The number of individuals analyzed in each case is indicated next to the upper whisker of each boxplot.

Journal of Cell Science • Supplementary information

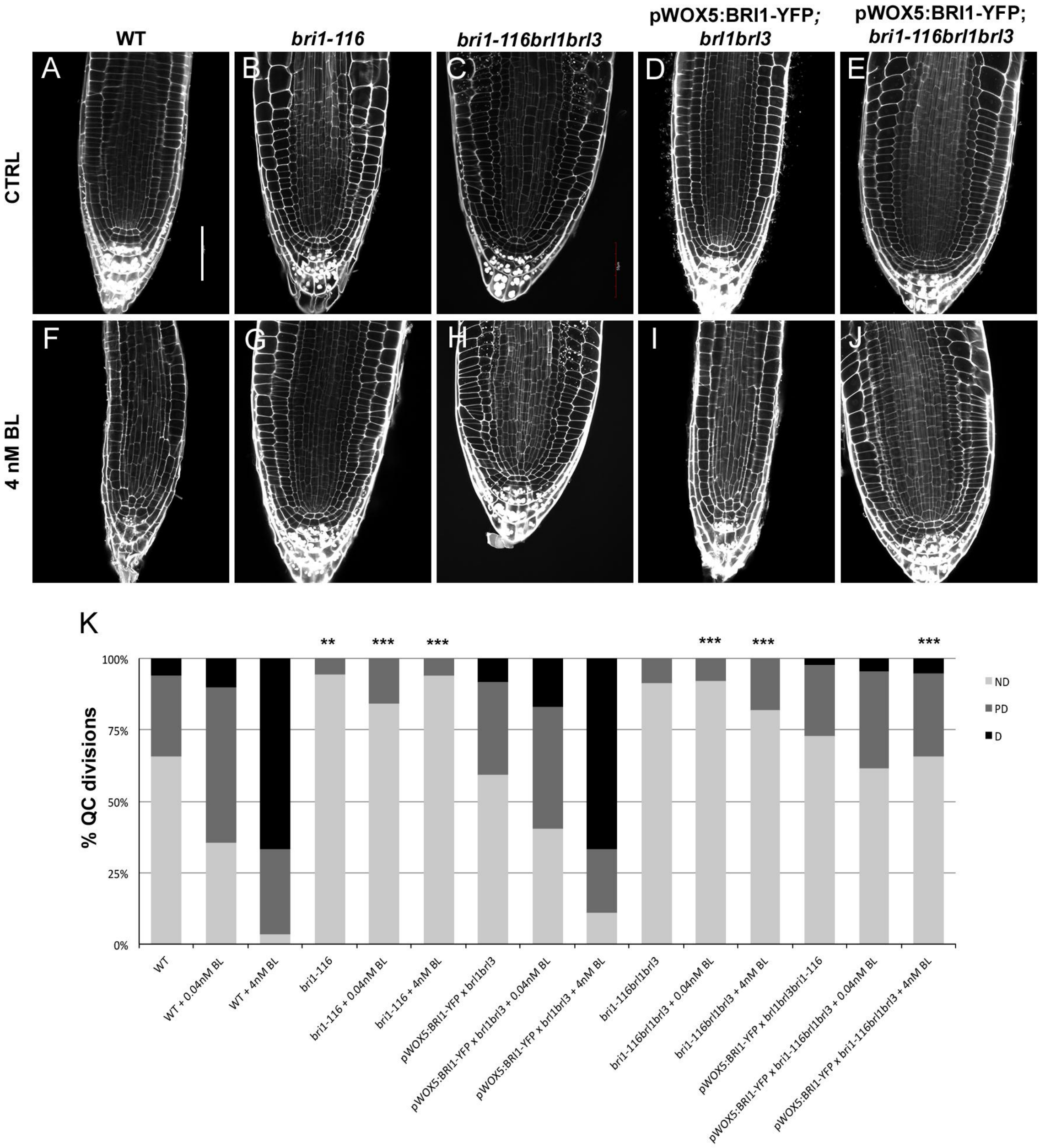

Figure S3. The BRL1 and BRL3 receptors do not compete with BRI1 for ligand binding in the QC microenvironment.

A-E) Phenotype of 6-day-old roots grown under control conditions. **F-J)** Phenotype of 6-day-old seedlings treated with BL. **K)** Quantification of QC division in control conditions, 0.04 nM BL or 4 nM of BL. Statistical differences in division rates were evaluated through a two-sided Fisher's test. Asterisks mean statistically significant differences respect to WT. Differences in division frequencies were assessed with a two-sided Fisher's test. Statistical values for all pairwise comparisons are provided in Supplementary Table S2. Data are generated from three independent replicates (n>34). ND = QC non-divided, PD = QC partially divided, D = QC totally divided. Scale bar: 50 µm.

Journal of Cell Science • Supplementary information

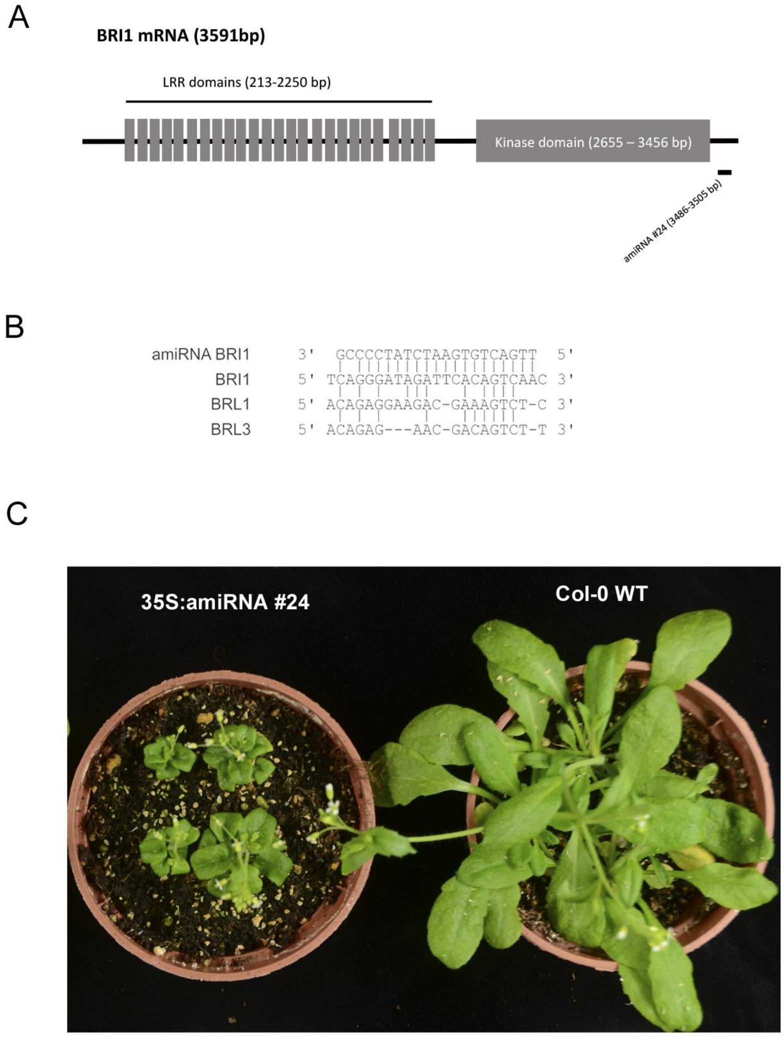

Figure S4. Generation of BRI1 amiRNA lines.

A) Schematic representation of the mRNA of BRI1 and the binding coordinates of the designed amiRNA. **B**) Sequence of the amiRNA and its base pairing with the mRNA of BRI1, BRL1 and BRL3. **C**) Images showing mature WT and 35S:BRI1-amiR#24 plants. The amiRNA#24 was chosen because its dwarf phenotype resembles the *bri1* mutant.

Journal of Cell Science • Supplementary information

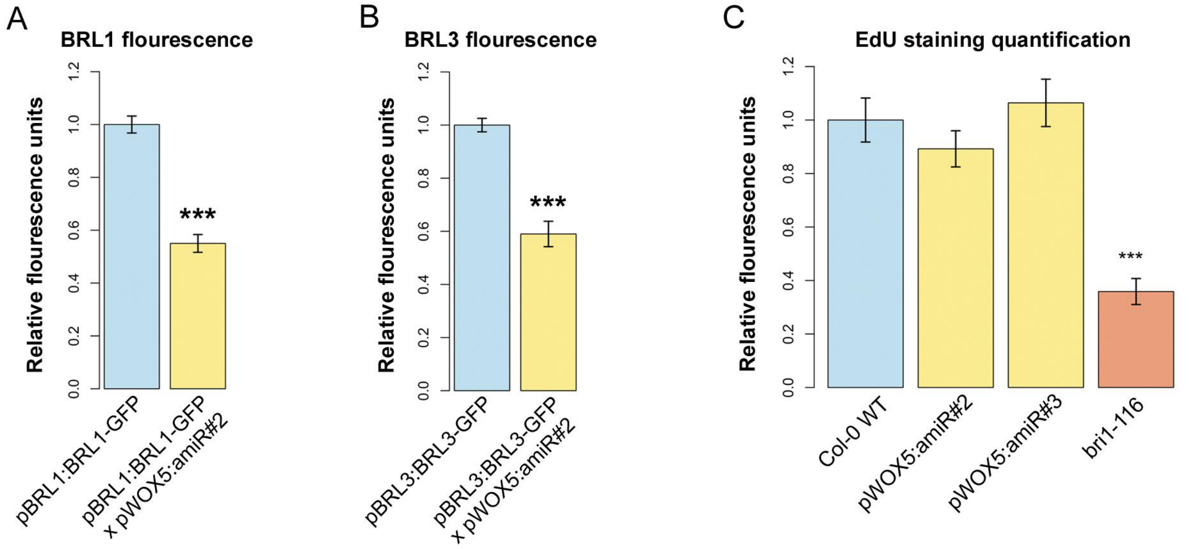

Figure S5. pWOX5:BRI1-amiR lines partially off-target BRL1 and BRL3 expression.

A-B) Fluorescence quantification of BRL1 and BRL3 proteins in the parental lines (pBRL1:BRL1-GFP, pBRL3:BRL3-GFP) and in the same lines crossed with the pWOX5:BRI1-amiR lines showed that they are downregulated as a consequence of the amiRNA activity. Asterisks mean statistically significant differences respect to WT in a two-tailed t-test. Data are generated from three independent replicates (n>22). **C)** Fluorescence quantification of the EdU incorporation shows no difference in cell division are found between the WT and pWOX5:BRI1-amiR lines. Asterisks mean statistically significant differences respect to WT in a two-tailed t-test. Data are generated from two independent replicates (n>15).

Table S1. Statistical analysis showing the p-values of two-sided Fisher's exact test for QC division rates in overexpressor lines

	WT	WT + BL	*bri1-116*	*bri1-116* + BL	pWOX5:BRI1-YFP	pWOX5:BRI1-YFP + BL	pWOX5:*bes1-D*-YFP	pWOX5:*bes1-D*-YFP + BL	pWOX5:BRI1-YFP x *bri1-116*	pWOX5:BRI1-YFP; *bri1-116* + BL	pWOX5:*bes1-D*-YFP; *bri1-116*	pWOX5:*bes1-D*-YFP; *bri1-116* + BL
**WT**	1	3.13E-41	0.065196943	0.645477605	0.002079891	1.80E-47	1.66E-47	2.79E-40	0.019180064	1.97E-09	7.90E-24	3.59E-35
**WT + BL**	3.13E-41	1	1.53E-44	3.30E-42	1.13E-29	0.303106278	0.132199029	1	2.19E-32	4.98E-18	1.02E-07	0.000804215
bri1-116	0.065196943	1.53E-44	1	0.250691643	3.77E-06	6.49E-50	2.46E-49	1.85E-43	8.25E-05	3.11E-13	2.47E-29	5.62E-41
***bri1-116* + BL**	0.645477605	3.30E-42	0.250691643	1	0.000410423	5.27E-48	4.40E-48	3.38E-41	0.004705678	2.42E-10	1.64E-25	4.09E-37
**pWOX5:BRI1-YFP**	0.002079891	1.13E-29	3.77E-06	0.000410423	1	9.27E-36	1.85E-36	9.76E-29	0.744905486	0.006703144	1.48E-12	2.88E-22
**pWOX5:BRI1-YFP + BL**	1.80E-47	0.303106278	6.49E-50	5.27E-48	9.27E-36	1	0.782676674	0.212427452	2.27E-38	1.34E-23	6.14E-12	2.01E-06
**pWOX5:*bes1-D*-YFP**	1.66E-47	0.132199029	2.46E-49	4.40E-48	1.85E-36	0.782676674	1	0.114255432	6.47E-39	1.81E-24	2.79E-13	8.64E-08
**pWOX5:*bes1-D*-YFP + BL**	2.79E-40	1	1.85E-43	3.38E-41	9.76E-29	0.212427452	0.114255432	1	3.11E-31	2.98E-17	2.11E-07	0.001098539
**pWOX5:BRI1-YFP x *bri1-116***	0.019180064	2.19E-32	8.25E-05	0.004705678	0.744905486	2.27E-38	6.47E-39	3.11E-31	1	0.000673097	1.06E-14	6.06E-25
**pWOX5:BRI1-YFP; *bri1-116* + BL**	1.97E-09	4.98E-18	3.11E-13	2.42E-10	0.006703144	1.34E-23	1.81E-24	2.98E-17	0.000673097	1	4.38E-05	8.80E-12
**pWOX5:*bes1-D*-YFP; *bri1-116***	7.90E-24	1.02E-07	2.47E-29	1.64E-25	1.48E-12	6.14E-12	2.79E-13	2.11E-07	1.06E-14	4.38E-05	1	0.01442438
**pWOX5:*bes1-D*-YFP; *bri1-116* + BL**	3.59E-35	0.000804215	5.62E-41	4.09E-37	2.88E-22	2.01E-06	8.64E-08	0.001098539	6.06E-25	8.80E-12	0.01442438	1

Journal of Cell Science • Supplementary information

Table S2. Statistical analysis showing the p-values of two-sided Fisher's exact test for QC division rates of overexpressor in triple mutant background

	**WT**	**WT + 0.04nM BL**	**WT + 4nM BL**	***bri1-116***	***bri1-116* + 0.04nM BL**	***bri1-116* + 4nM BL**	**pWOX5:BRI1-YFP x *brl1brl3***	**pWOX5:BRI1-YFP x *brl1brl3* + 0.04nM BL**	**pWOX5:BRI1-YFP x *brl1brl3* + 4nM BL**	***bri1brl1brl3***	***bri1brl1brl3* + 0.04nM BL**	***bri1brl1brl3* + 4nM BL**	**pWOX5:BRI1-YFP x *bri1brl1brl3***	**pWOX5:BRI1-YFP x *bri1brl1brl3* + 0.04nM BL**	**pWOX5:BRI1-YFP x *bri1brl1brl3* + 4nM BL**
**WT**	1.00E+00	2.22E-04	4.87E-21	1.99E-03	4.67E-02	2.52E-04	6.60E-01	8.09E-03	1.03E-13	1.34E-02	6.45E-03	1.30E-01	6.25E-01	8.24E-01	1.00E+00
**WT + 0.04nM BL**	2.22E-04	1.00E+00	1.20E-12	2.72E-09	3.04E-07	1.39E-11	2.93E-02	3.40E-01	4.88E-09	5.47E-08	1.37E-08	3.70E-06	3.35E-04	2.23E-02	8.22E-03
**WT + 4nM BL**	4.87E-21	1.20E-12	1.00E+00	1.44E-21	8.22E-21	7.39E-26	2.14E-13	2.12E-08	2.88E-01	6.46E-20	4.19E-21	3.88E-19	2.65E-17	3.07E-14	1.12E-13
bri1-116	1.99E-03	2.72E-09	1.44E-21	1.00E+00	1.75E-01	1.00E+00	4.98E-04	6.36E-07	1.63E-14	6.69E-01	1.00E+00	1.56E-01	2.28E-02	1.00E-03	4.90E-03
***bri1-116* + 0.04nM BL**	4.67E-02	3.04E-07	8.22E-21	1.75E-01	1.00E+00	1.79E-01	1.39E-02	1.35E-05	1.87E-14	5.00E-01	3.32E-01	1.00E+00	3.00E-01	3.64E-02	8.34E-02
***bri1-116* + 4nM BL**	2.52E-04	1.39E-11	7.39E-26	1.00E+00	1.79E-01	1.00E+00	5.04E-05	8.80E-09	1.58E-17	6.79E-01	6.93E-01	9.47E-02	5.72E-03	1.72E-04	1.07E-03
**pWOX5:BRI1-YFP x *brl1brl3***	6.60E-01	2.93E-02	2.14E-13	4.98E-04	1.39E-02	5.04E-05	1.00E+00	1.48E-01	8.56E-09	3.85E-03	1.60E-03	3.35E-02	2.89E-01	8.43E-01	8.33E-01
**pWOX5:BRI1-YFP x *brl1brl3* + 0.04nM BL**	8.09E-03	3.40E-01	2.12E-08	6.36E-07	1.35E-05	8.80E-09	1.48E-01	1.00E+00	1.61E-05	3.40E-06	1.48E-06	8.86E-05	3.20E-03	5.87E-02	5.39E-02
**pWOX5:BRI1-YFP x *brl1brl3* + 4nM BL**	1.03E-13	4.88E-09	2.88E-01	1.63E-14	1.87E-14	1.58E-17	8.56E-09	1.61E-05	1.00E+00	1.71E-13	2.47E-14	2.83E-13	1.08E-11	9.72E-10	3.72E-09
bri1brl1brl3	1.34E-02	5.47E-08	6.46E-20	6.69E-01	5.00E-01	6.79E-01	3.85E-03	3.40E-06	1.71E-13	1.00E+00	1.00E+00	3.21E-01	1.02E-01	4.28E-03	2.03E-02
***bri1brl1brl3* + 0.04nM BL**	6.45E-03	1.37E-08	4.19E-21	1.00E+00	3.32E-01	6.93E-01	1.60E-03	1.48E-06	2.47E-14	1.00E+00	1.00E+00	3.11E-01	5.81E-02	2.74E-03	1.16E-02
***bri1brl1brl3* + 4nM BL**	1.30E-01	3.70E-06	3.88E-19	1.56E-01	1.00E+00	9.47E-02	3.35E-02	8.86E-05	2.83E-13	3.21E-01	3.11E-01	1.00E+00	5.11E-01	6.32E-02	1.41E-01
**pWOX5:BRI1-YFP x *bri1brl1brl3***	6.25E-01	3.35E-04	2.65E-17	2.28E-02	3.00E-01	5.72E-03	2.89E-01	3.20E-03	1.08E-11	1.02E-01	5.81E-02	5.11E-01	1.00E+00	5.09E-01	7.10E-01
**pWOX5:BRI1-YFP x *bri1brl1brl3* + 0.04nM BL**	8.24E-01	2.23E-02	3.07E-14	1.00E-03	3.64E-02	1.72E-04	8.43E-01	5.87E-02	9.72E-10	4.28E-03	2.74E-03	6.32E-02	5.09E-01	1.00E+00	9.28E-01
**pWOX5:BRI1-YFP x *bri1brl1brl3* + 4nM BL**	1.00E+00	8.22E-03	1.12E-13	4.90E-03	8.34E-02	1.07E-03	8.33E-01	5.39E-02	3.72E-09	2.03E-02	1.16E-02	1.41E-01	7.10E-01	9.28E-01	1.00E+00

Table S3. Statistical analysis showing the p-values of two-sided Fisher's exact test for QC division rates in pWOX5:BRI1-amiR lines

	**WT**	**WT + BL**	**pWOX5:amiR #2**	**pWOX5:amiR #2 + BL**	**pWOX5:amiR #3**	**pWOX5:amiR #3 + BL**
**WT**	1	3.41E-14	0.134284396	0.457724433	0.223998693	0.347302207
**WT + BL**	3.41E-14	1	2.52E-20	2.84E-18	3.58E-20	4.14E-17
**pWOX5:amiR #2**	0.134284396	2.52E-20	1	0.454826751	0.803164383	0.096876118
**pWOX5:amiR #2 + BL**	0.457724433	2.84E-18	0.454826751	1	0.632179749	0.490367755
**pWOX5:amiR #3**	0.223998693	3.58E-20	0.803164383	0.632179749	1	0.173022885
**pWOX5:amiR #3 + BL**	0.347302207	4.14E-17	0.096876118	0.490367755	0.173022885	1

Table S4. Statistical analysis showing the p-values of two-sided Fisher's exact test for quantification of vascular cell death after 24h of bleomycin treatment

	**WT**	**pWOX5:amiR #2**	**pWOX5:amiR #3**	***bri1-116***
**WT**	1	0.523755383	0.936521696	2.17E-06
**pWOX5:amiR #3**	0.523755383	1	0.736002896	5.24E-08
**pWOX5:amiR #2**	0.936521696	0.736002896	1	9.14E-07
bri1-116	2.17E-06	5.24E-08	9.14E-07	1

Table S5. Statistical analysis showing the p-values of two-sided Fisher's exact test for QC division frequencies upon DNA damage

	**WT**	**pWOX5:amiR #2**	**pWOX5:amiR #3**	***bri1-116***
**WT**	1	0.003715706	0.005890947	0.000181417
**pWOX5:amiR #2**	0.003715706	1	0.882973236	0.344123125
**pWOX5:amiR #3**	0.005890947	0.882973236	1	0.480118388
bri1-116	0.000181417	0.344123125	0.480118388	1

Table S6. Plant lines used in this work

Name	Description	Comments	Reference
bri1-116	BR-signalling	Knock out	Li and Chory, 1997
brl1	BR-signalling	Knock out	Caño-Delgado et al., 2004
brl3	BR-signalling	Knock out	Caño-Delgado et al., 2004
Bes1-D	BR-signalling	Gain-of-function	Yin et al., 2002
pWOX5:BRI1-YFP	Translational fusion	QC overexpression	This work
pWOX5:*bes1-D*-YFP	Translational fusion	QC overexpression	Vilarrasa-Blasi et al., 2014
pSCR:BRI1-YFP	Translational fusion	Endodermis overexpression	Hacham et al., 2011
pBRL1:BRL1-YFP	Translational fusion	BRL1 expression marker	Fàbregas et al., 2013
pBRL3:BRL3-YFP	Translational fusion	BRL3 expression marker	Fàbregas et al., 2013
pWOX5:amiRNA	Transcriptional fusion	Tissue-specific BRI1 knock down	This work

Journal of Cell Science • Supplementary information
